# Supplementary material for: Origins and diversity of pan-isotype human bone marrow plasma cells
Source: bioRxiv. 2024 May 10:2024.05.08.592267. Preprint. [Version 1] doi: 10.1101/2024.05.08.592267 (PMC11100731; doi:10.1101/2024.05.08.592267)
Supplement: Supplement 1 [file NIHPP2024.05.08.592267v1-supplement-1.pdf]

bioRxiv preprint doi: https://doi.org/10.1101/2024.05.08.592267; this version posted May 10, 2024. The copyright holder for this preprint (which was not certified by peer review) is the author/funder, who has granted bioRxiv a license to display the preprint in perpetuity. It is made available under aCC-BY 4.0 International license.

Supplementary Figures

bioRxiv preprint doi: https://doi.org/10.1101/2024.05.08.592267; this version posted May 10, 2024. The copyright holder for this preprint (which was not certified by peer review) is the author/funder, who has granted bioRxiv a license to display the preprint in perpetuity. It is made available under aCC-BY 4.0 International license.

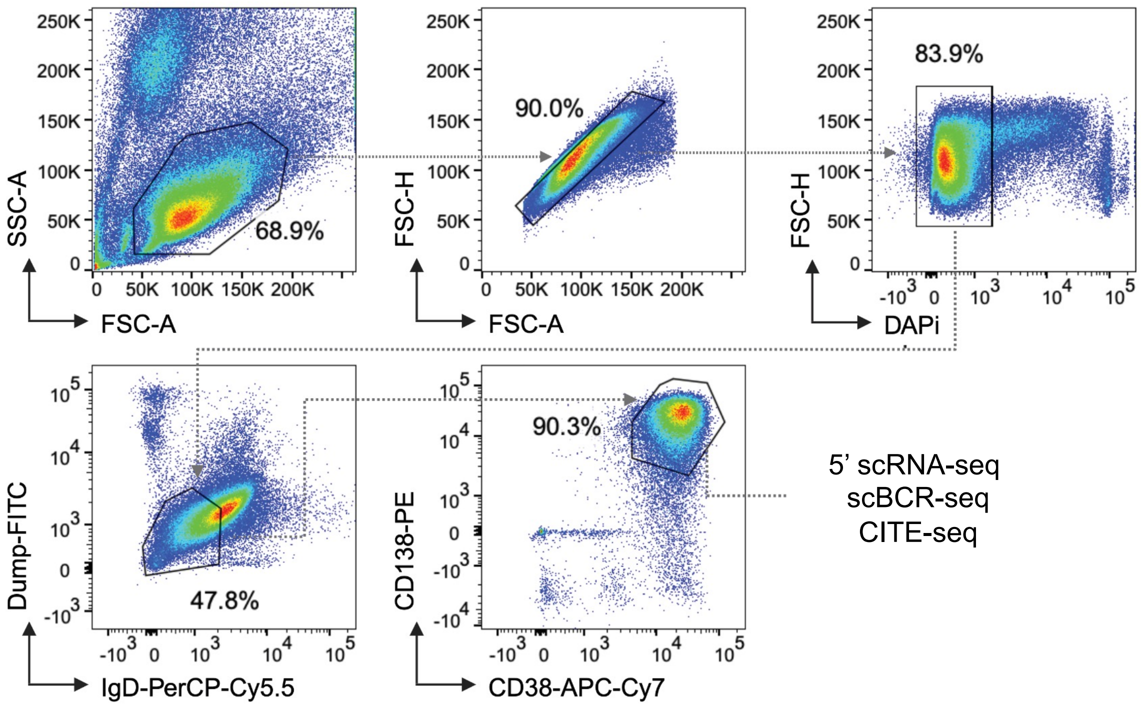

bioRxiv preprint doi: https://doi.org/10.1101/2024.05.08.592267; this version posted May 10, 2024. The copyright holder for this preprint (which was not certified by peer review) is the author/funder, who has granted bioRxiv a license to display the preprint in perpetuity. It is made available under aCC-BY 4.0 International license.

Figure S1. Gating strategy.

FACS sorting of magnetically enriched $CD138^+$ human bone marrow plasma cells. Dump channel contains anti-CD3 and anti-CD14 antibodies.

bioRxiv preprint doi: https://doi.org/10.1101/2024.05.08.592267; this version posted May 10, 2024. The copyright holder for this preprint (which was not certified by peer review) is the author/funder, who has granted bioRxiv a license to display the preprint in perpetuity. It is made available under aCC-BY 4.0 International license.

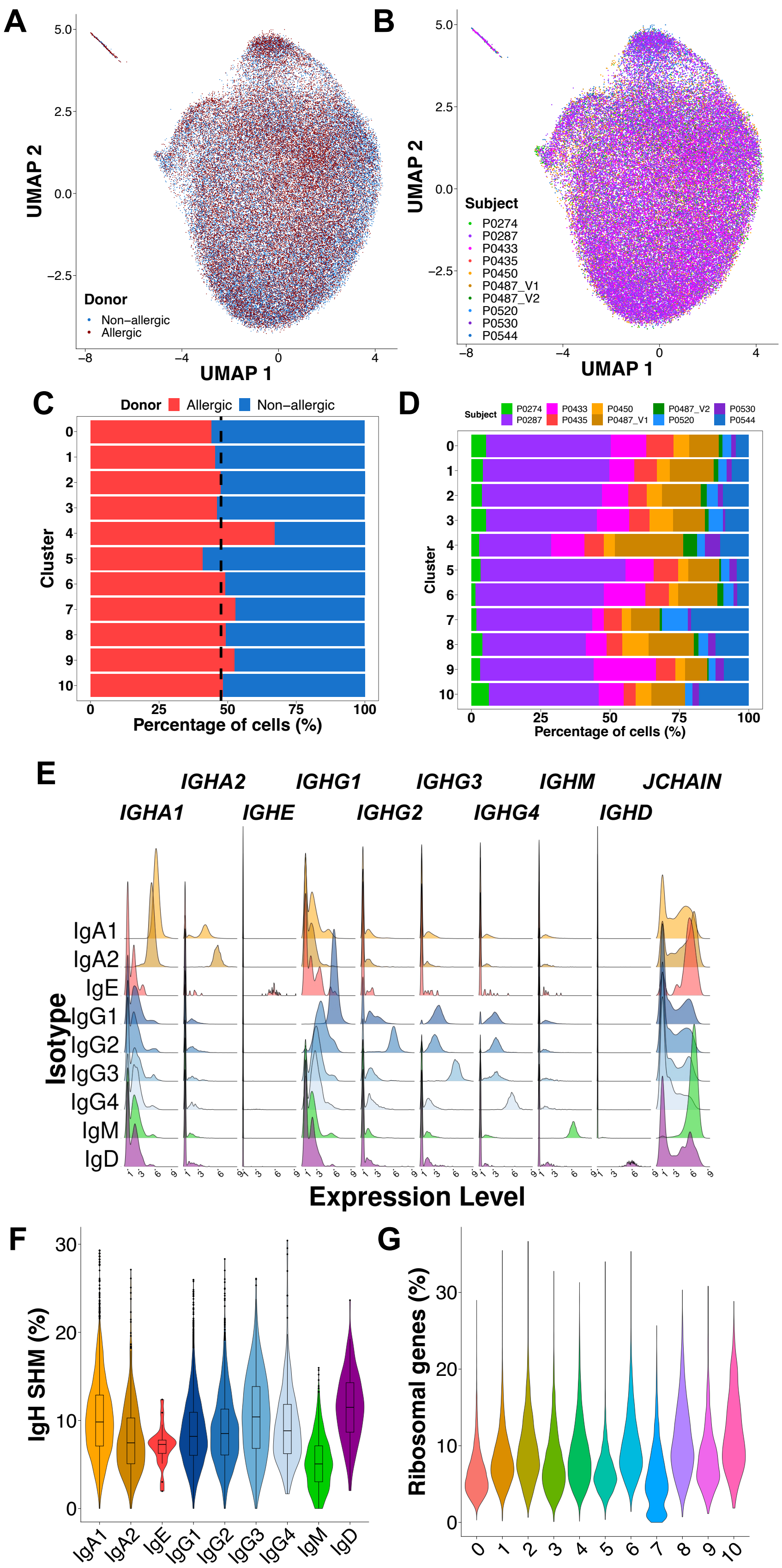

bioRxiv preprint doi: https://doi.org/10.1101/2024.05.08.592267; this version posted May 10, 2024. The copyright holder for this preprint (which was not certified by peer review) is the author/funder, who has granted bioRxiv a license to display the preprint in perpetuity. It is made available under aCC-BY 4.0 International license.

Figure S2. Quality control of clustered cells.

(A) UMAP projection of BMPCs, colored by donor's allergy status.

(B) Proportions of cells in each cluster by donor's allergy status. Black dashed line represents expected proportion based on input number of cells per allergy status (n = 34,991 for allergic, n = 38,569 for non-allergic).

(C) UMAP projection of BMPCs, colored by donor.

(D) Proportions of cells in each cluster by donor.

(E) Ridge plots for IgH gene expression on BMPCs of different IgH isotypes.

(F) Heavy chain somatic hypermutation (IgH SHM) percentage of BMPCs of different IgH isotypes.

(G) Percentage of ribosomal genes in the transcriptome of each cluster.

bioRxiv preprint doi: https://doi.org/10.1101/2024.05.08.592267; this version posted May 10, 2024. The copyright holder for this preprint (which was not certified by peer review) is the author/funder, who has granted bioRxiv a license to display the preprint in perpetuity. It is made available under aCC-BY 4.0 International license.

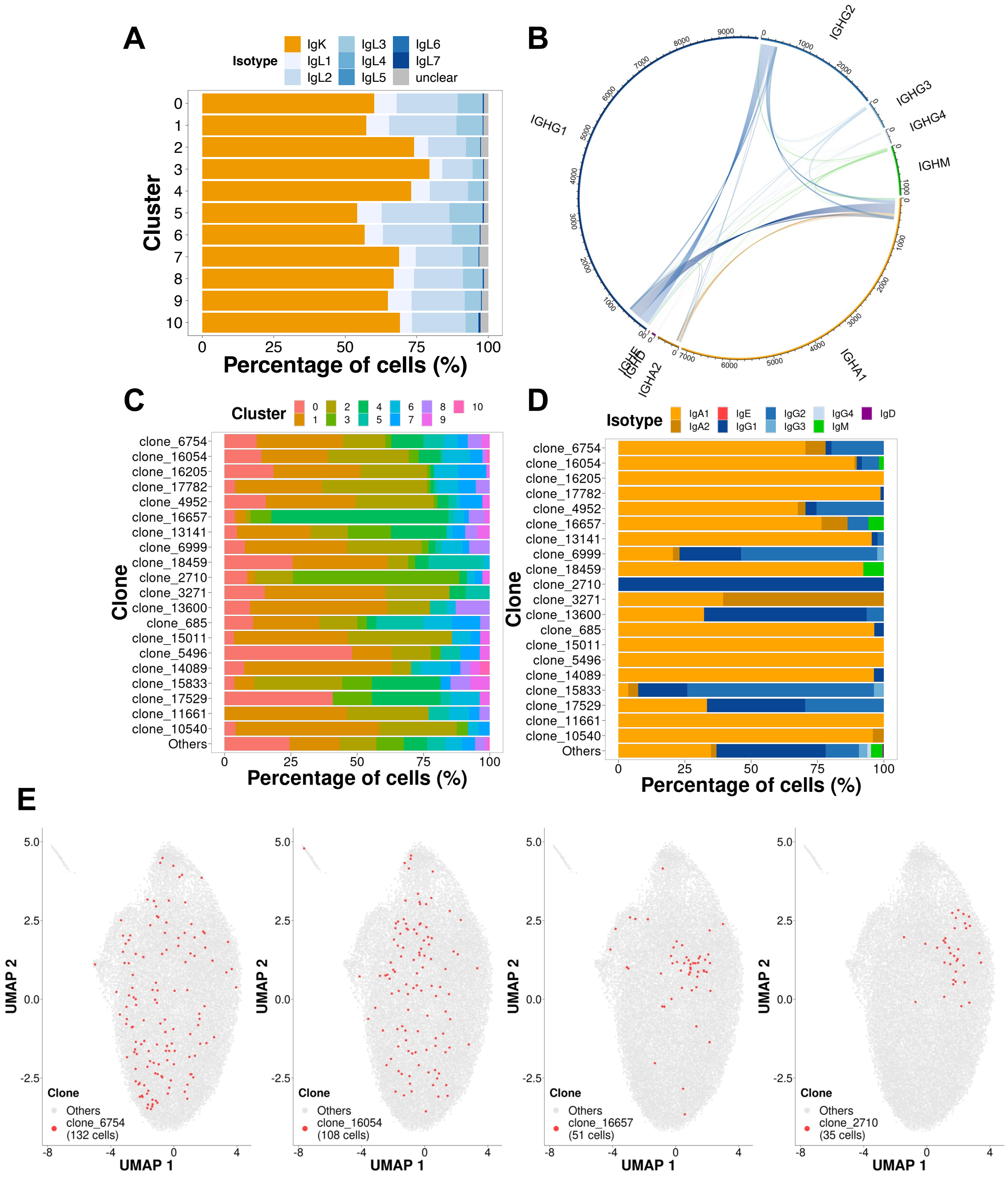

bioRxiv preprint doi: https://doi.org/10.1101/2024.05.08.592267; this version posted May 10, 2024. The copyright holder for this preprint (which was not certified by peer review) is the author/funder, who has granted bioRxiv a license to display the preprint in perpetuity. It is made available under aCC-BY 4.0 International license.

Figure S3.

(A) Proportion of cells in each cluster by IgL isotype.

(B) Clonal relationships between BMPCs of different isotypes. Lines connect cells that have the same IgH V and J segments, and over 82% identity in CDRH3 (Hamming distance determined by Shazam = 0.18). Line end width is proportional to the number of cells in the clonal family.

(C-D) Top 20 most expanded clonal families. Composition of each family is colored by cluster identity (C) and IgH isotype (D). Remaining aggregated BMPCs are included for reference ("Others").

(E) UMAP projection of clustered BMPCs. Largest clone families (6754 & 16054) and predominantly mono-isotypic clone families (16657 & 2710) showed in **(C)** and **(D)** are highlighted in red.

bioRxiv preprint doi: https://doi.org/10.1101/2024.05.08.592267; this version posted May 10, 2024. The copyright holder for this preprint (which was not certified by peer review) is the author/funder, who has granted bioRxiv a license to display the preprint in perpetuity. It is made available under aCC-BY 4.0 International license.

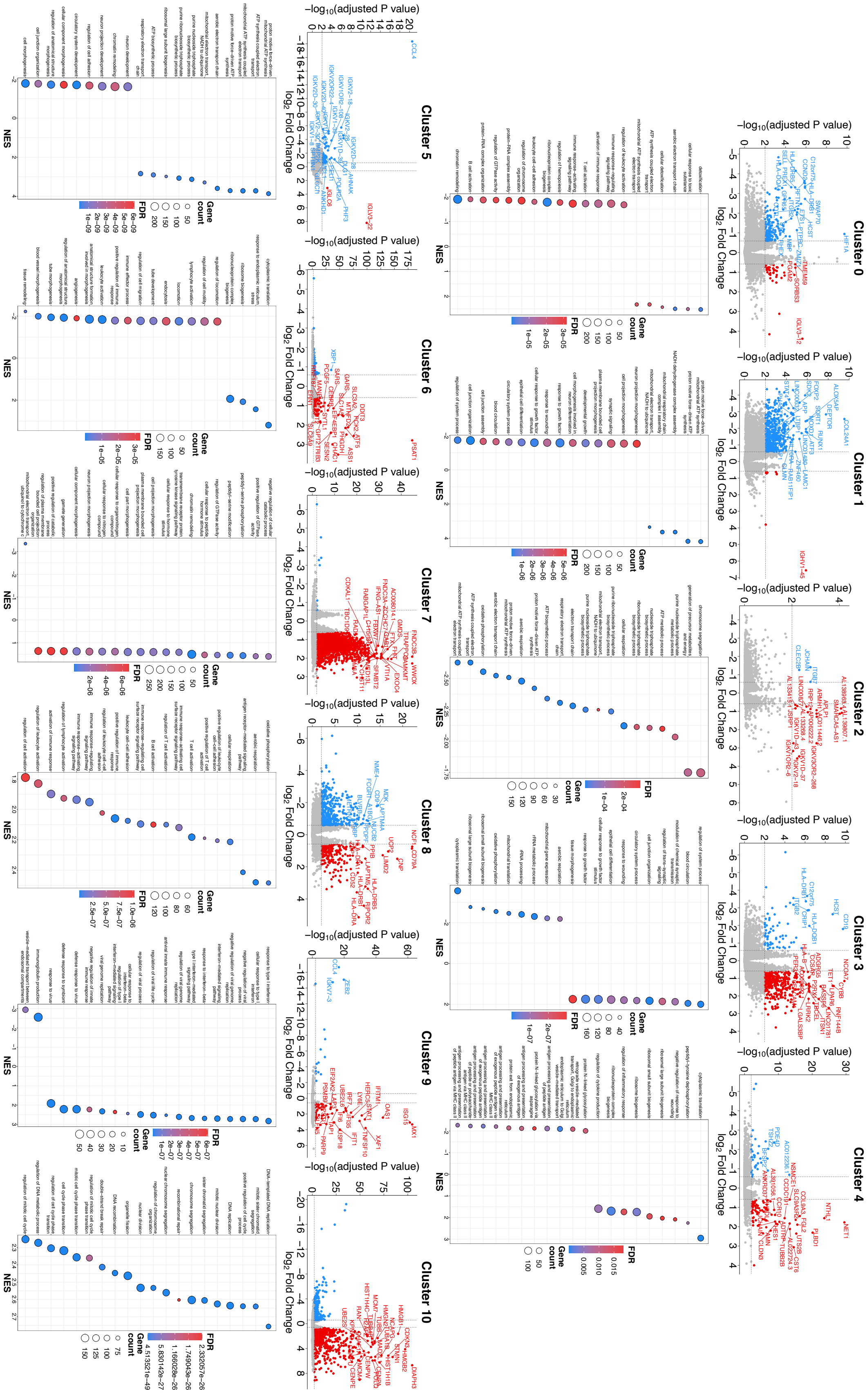

bioRxiv preprint doi: https://doi.org/10.1101/2024.05.08.592267; this version posted May 10, 2024. The copyright holder for this preprint (which was not certified by peer review) is the author/funder, who has granted bioRxiv a license to display the preprint in perpetuity. It is made available under aCC-BY 4.0 International license.

Figure S4. Pathway analysis for clusters of BMPCs.

Differential expression gene testing was performed on BMPCs pseudobulked by donor and cluster identity. For each set of genes, gene set enrichment analysis was performed. Volcano plots and the top 20 GSEA results by false discovery rate (FDR) are shown for each cluster.

bioRxiv preprint doi: https://doi.org/10.1101/2024.05.08.592267; this version posted May 10, 2024. The copyright holder for this preprint (which was not certified by peer review) is the author/funder, who has granted bioRxiv a license to display the preprint in perpetuity. It is made available under aCC-BY 4.0 International license.

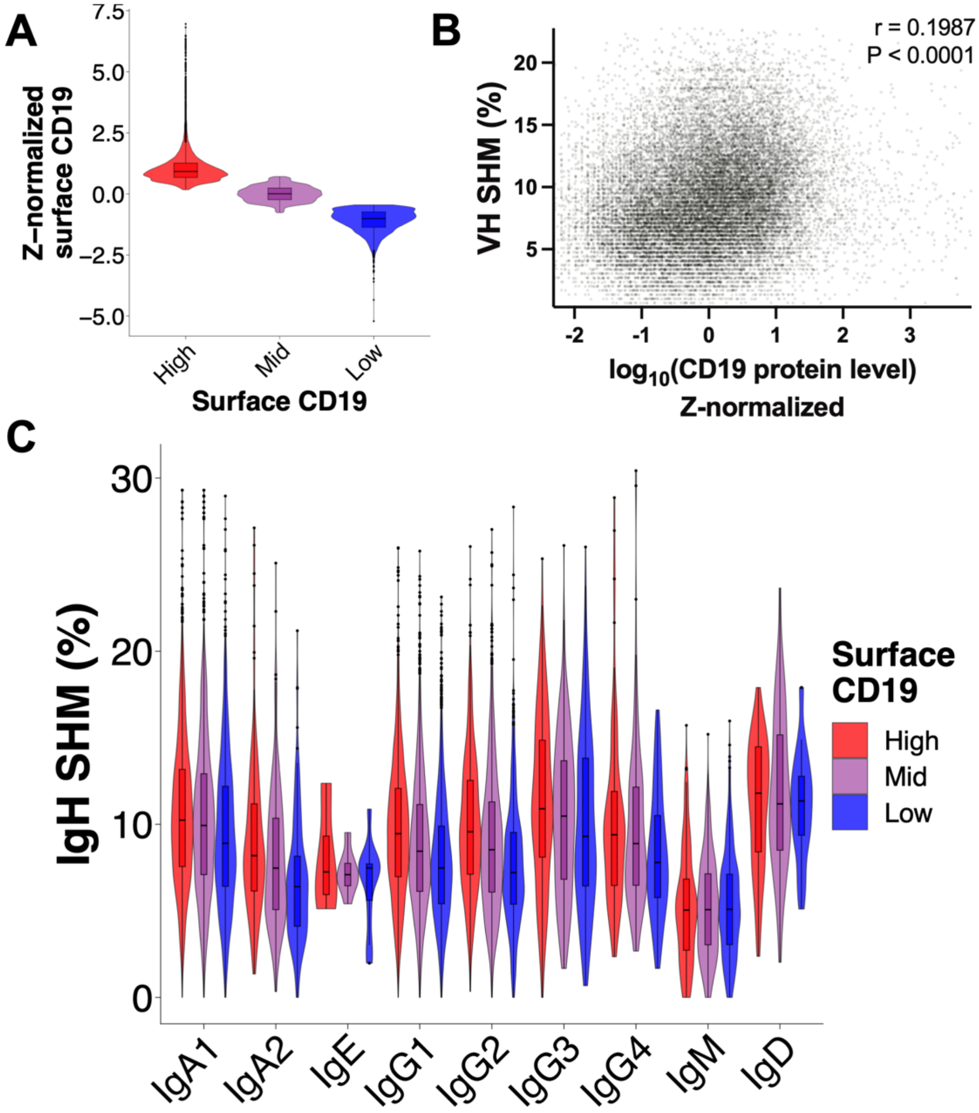

bioRxiv preprint doi: https://doi.org/10.1101/2024.05.08.592267; this version posted May 10, 2024. The copyright holder for this preprint (which was not certified by peer review) is the author/funder, who has granted bioRxiv a license to display the preprint in perpetuity. It is made available under aCC-BY 4.0 International license.

Figure S5. Surface CD19 expression and SHM burden are correlated.

(A) Normalized surface CD19 expression on CD19 expression categories among BMPCs.

(B) Correlation between normalized surface CD19 expression and IgH SHM (n = 23,289). Spearman correlation was performed. 99.5% of the distribution in the X axis and 99.5% of the distribution in the Y axis are shown.

(C) IgH SHM of BMPCs of different isotypes, subdivided by surface CD19 expression categories. Sample sizes for CD19 high/mid/low are: IgA1 = 3,137 / 3,497 / 1,892; IgA2 = 165 / 218 / 141; IgE = 4 / 8 / 10; IgG1 = 2,137 / 3,411 / 3,401; IgG2 = 926 / 1,188 / 760; IgG3 = 160 / 295 / 230; IgG4 = 103 / 128 / 73; IgM = 252 / 485 / 457; IgD = 42 / 41 /30).

bioRxiv preprint doi: https://doi.org/10.1101/2024.05.08.592267; this version posted May 10, 2024. The copyright holder for this preprint (which was not certified by peer review) is the author/funder, who has granted bioRxiv a license to display the preprint in perpetuity. It is made available under aCC-BY 4.0 International license.

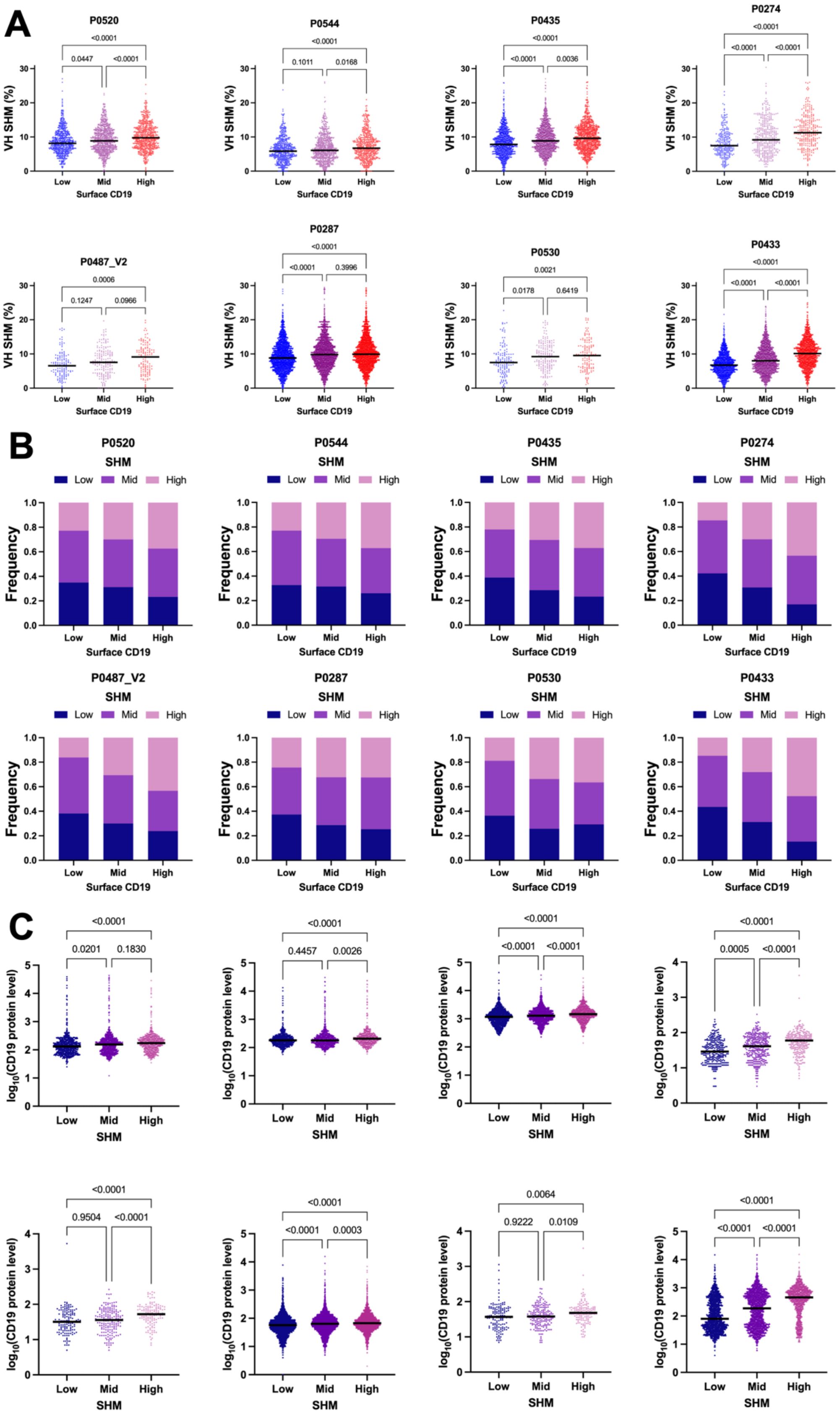

bioRxiv preprint doi: https://doi.org/10.1101/2024.05.08.592267; this version posted May 10, 2024. The copyright holder for this preprint (which was not certified by peer review) is the author/funder, who has granted bioRxiv a license to display the preprint in perpetuity. It is made available under aCC-BY 4.0 International license.

Figure S6. Features indicative of BMPC origin are retained at the donor level.

(A) IgH SHM per surface CD19 expression bins per donor. Kruskal-Wallis test was used to assess statistical significance.

(B) Frequency of IgH SHM bins per cluster per donor.

(C) Surface CD19 expression per IgH SHM bins per donor. Kruskal-Wallis test was used to assess statistical significance.

bioRxiv preprint doi: https://doi.org/10.1101/2024.05.08.592267; this version posted May 10, 2024. The copyright holder for this preprint (which was not certified by peer review) is the author/funder, who has granted bioRxiv a license to display the preprint in perpetuity. It is made available under aCC-BY 4.0 International license.

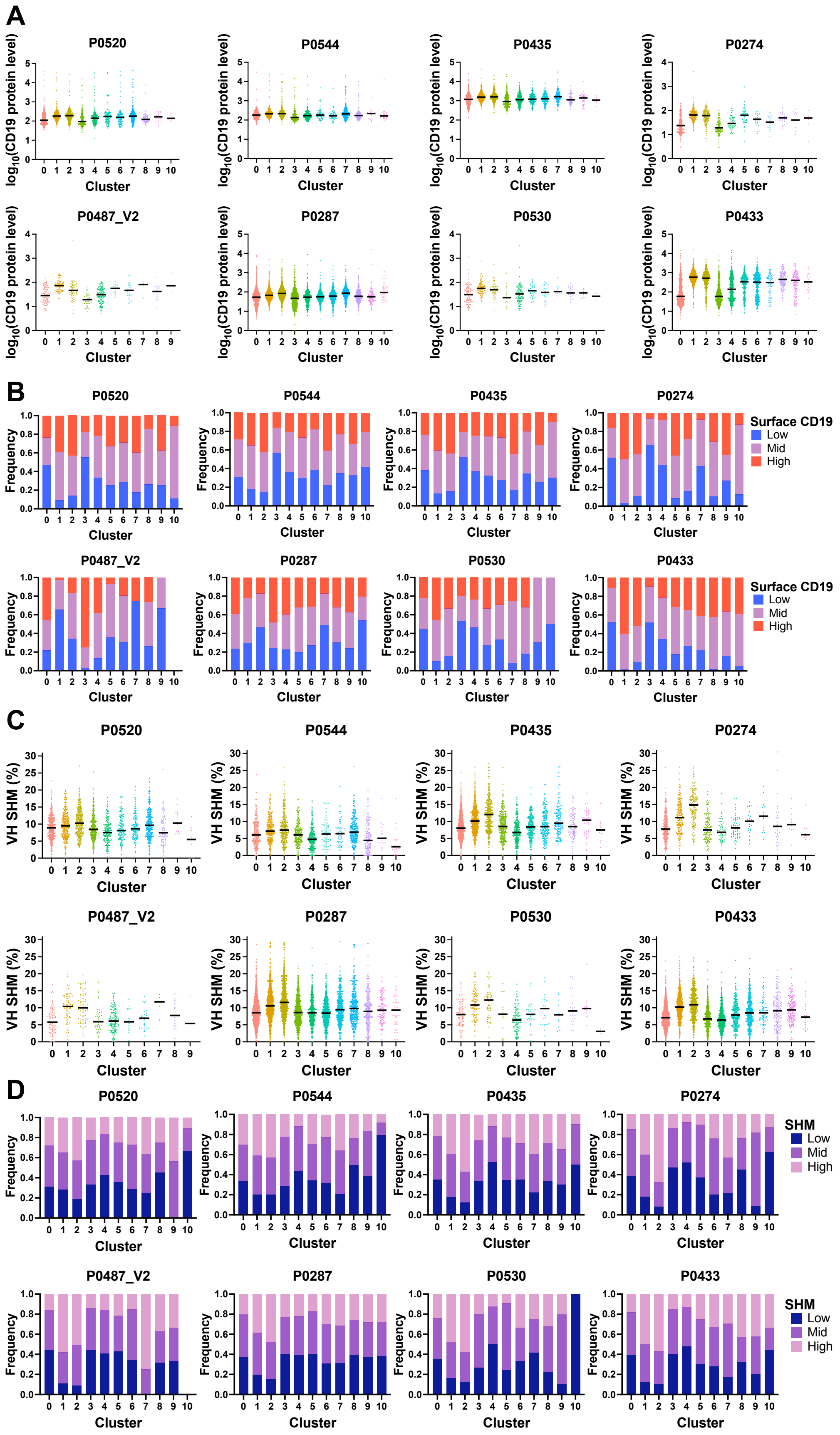

bioRxiv preprint doi: https://doi.org/10.1101/2024.05.08.592267; this version posted May 10, 2024. The copyright holder for this preprint (which was not certified by peer review) is the author/funder, who has granted bioRxiv a license to display the preprint in perpetuity. It is made available under aCC-BY 4.0 International license.

Figure S7. Surface CD19 expression and SHM levels are retained at the donor level.

(A) Surface CD19 expression on BMPCs per cluster per donor.

(B) Frequency of surface CD19 expression bins per cluster per donor.

(C) Somatic hypermutation (SHM) in IgH chain per cluster per donor.

(D) Frequency of IgH SHM bins per surface CD19 expression bins per donor.

bioRxiv preprint doi: https://doi.org/10.1101/2024.05.08.592267; this version posted May 10, 2024. The copyright holder for this preprint (which was not certified by peer review) is the author/funder, who has granted bioRxiv a license to display the preprint in perpetuity. It is made available under aCC-BY 4.0 International license.

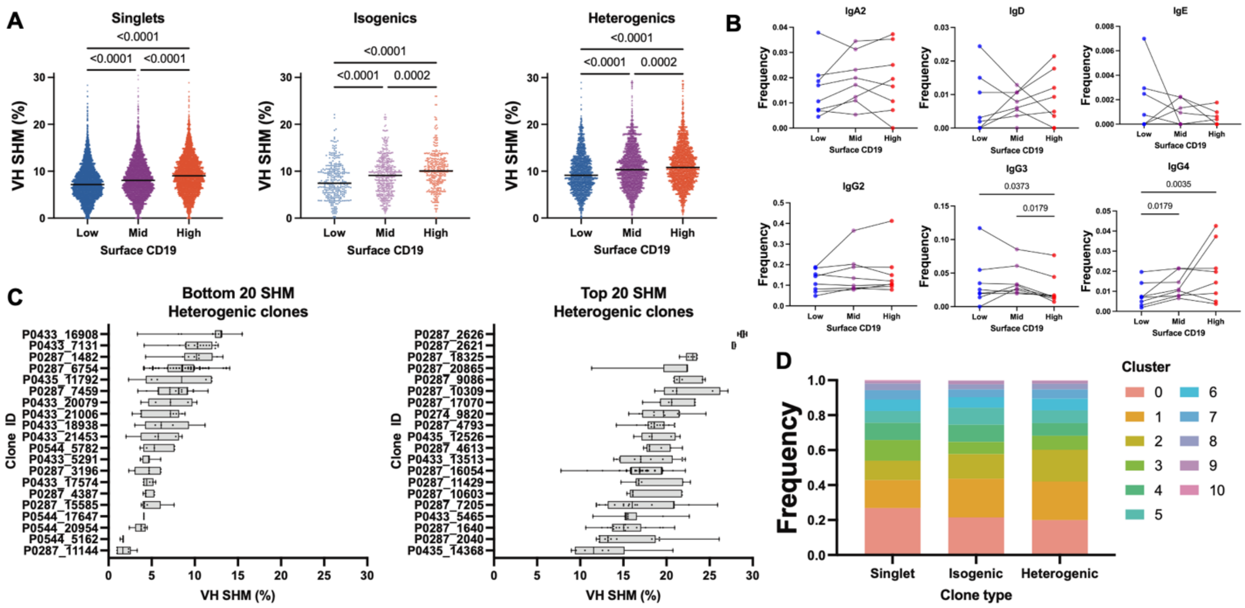

bioRxiv preprint doi: https://doi.org/10.1101/2024.05.08.592267; this version posted May 10, 2024. The copyright holder for this preprint (which was not certified by peer review) is the author/funder, who has granted bioRxiv a license to display the preprint in perpetuity. It is made available under aCC-BY 4.0 International license.

Figure S8. Features of clonal structure related to BMPC origins.

(A) IgH somatic hypermutation (SHM) per surface CD19 bin in clonal families with different clonal structures. Kruskal-Wallis test was used to assess statistical significance.

(B) Frequency of surface CD19 bins among IgA2, IgD, IgE, and IgG2/3/4 BMPCs. Lines connecting dots represent an individual donor. Friedman test was used to assess statistical significance. Only statistically significant differences are shown.

(C) IgH SHM in the 20 least mutated and the 20 most mutated heterogenic clones (related to Fig. 3I).

(D) Frequency of cluster identities among clonal families with different clonal structures.

bioRxiv preprint doi: https://doi.org/10.1101/2024.05.08.592267; this version posted May 10, 2024. The copyright holder for this preprint (which was not certified by peer review) is the author/funder, who has granted bioRxiv a license to display the preprint in perpetuity. It is made available under aCC-BY 4.0 International license.

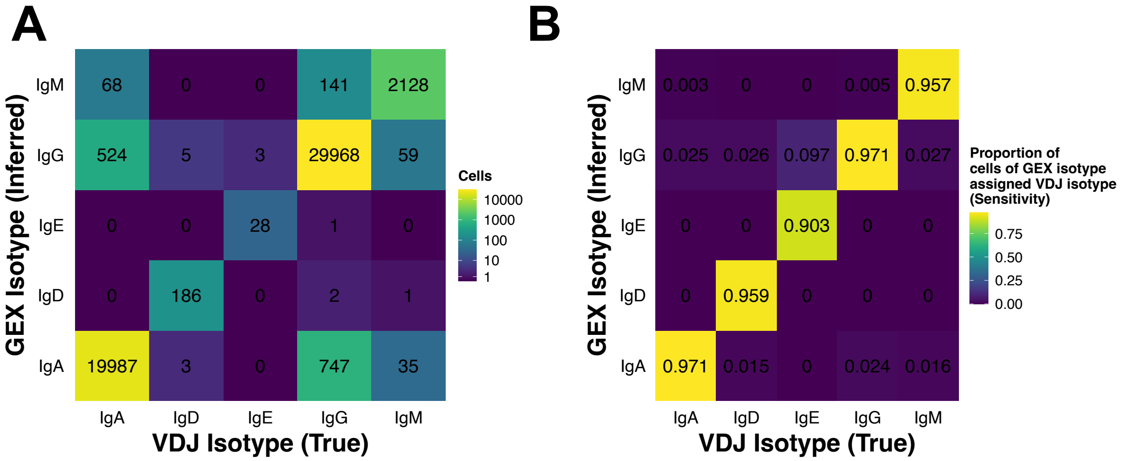

C

Isotype	FPR (%)	FNR (%)	Sensitivity (%)	Specificity (%)	Accuracy (%)
**IgA**	2.36	2.88	97.12	97.64	97.44
**IgD**	0.01	4.12	95.88	99.99	99.98
**IgE**	0.00	9.68	90.32	99.99	99.99
**IgG**	2.57	2.89	97.11	97.43	99.44
**IgM**	0.40	4.27	95.73	99.60	99.49

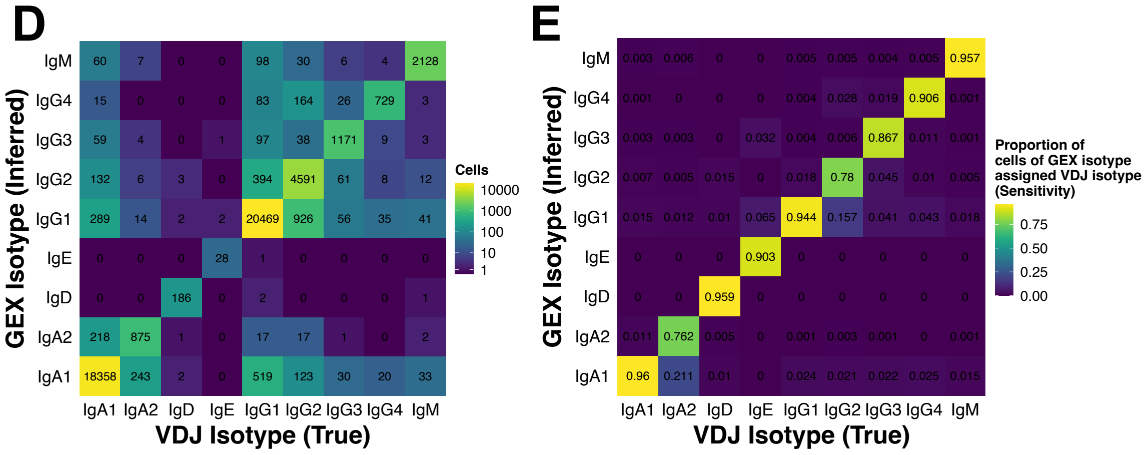

F

Isotype	FPR (%)	FNR (%)	Sensitivity (%)	Specificity (%)	Accuracy (%)
**IgA1**	2.91	4.04	95.96	97.09	96.68
**IgA2**	0.00	23.85	76.15	99.50	98.99
**IgD**	0.01	4.12	95.88	99.99	99.98
**IgE**	0.00	9.68	90.32	99.99	99.99
**IgG1**	4.44	5.59	94.41	95.56	95.09
**IgG2**	1.32	22.04	77.96	98.68	96.35
**IgG3**	0.41	13.32	86.68	99.59	99.25
**IgG4**	0.56	9.44	90.56	99.44	99.30
**IgM**	0.41	4.27	95.73	99.59	99.43

bioRxiv preprint doi: https://doi.org/10.1101/2024.05.08.592267; this version posted May 10, 2024. The copyright holder for this preprint (which was not certified by peer review) is the author/funder, who has granted bioRxiv a license to display the preprint in perpetuity. It is made available under aCC-BY 4.0 International license.

Figure S9. Performance of GEX-based isotype calling when benchmarked against VDJ-based isotype calling.

(A) Number of cells assigned IgH isotype class based on gene expression (GEX) library, compared to "true" isotype class calling based on VDJ library (n = 49,667).

(B) Percentage of cells of a given IgH isotype class (as determined by GEX library) that are inferred to pertain to each IgH isotype class based on VDJ library (n = 49,667).

(C) Performance metrics of GEX-based isotype calling when benchmarked against VDJ library callings at the IgH class level. FPR = False positive rate. FNR = False negative rate.

(D) Number of cells assigned IgH isotype subclass based on GEX library, compared to "true" isotype subclass calling based on VDJ library (n = 49,667).

(E) Percentage of cells of a given IgH isotype subclass (as determined by GEX library) that are inferred to pertain to each IgH isotype subclass based on VDJ library (n = 49,667).

(F) Performance metrics of GEX-based isotype calling when benchmarked against VDJ library callings at the IgH subclass level. FPR = False positive rate. FNR = False negative rate.

bioRxiv preprint doi: https://doi.org/10.1101/2024.05.08.592267; this version posted May 10, 2024. The copyright holder for this preprint (which was not certified by peer review) is the author/funder, who has granted bioRxiv a license to display the preprint in perpetuity. It is made available under aCC-BY 4.0 International license.

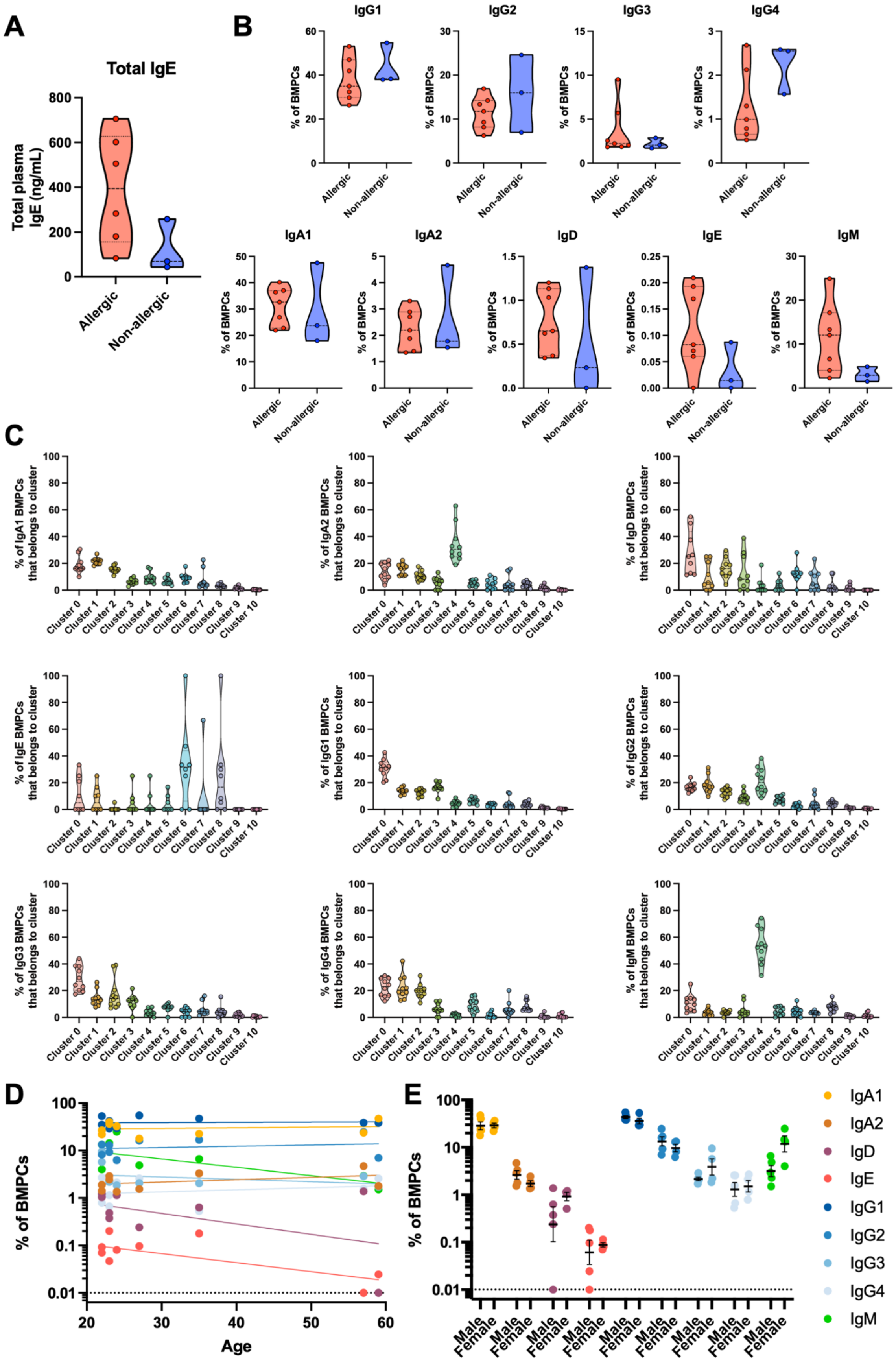

bioRxiv preprint doi: https://doi.org/10.1101/2024.05.08.592267; this version posted May 10, 2024. The copyright holder for this preprint (which was not certified by peer review) is the author/funder, who has granted bioRxiv a license to display the preprint in perpetuity. It is made available under aCC-BY 4.0 International license.

Fig. S10. Plasma cell heavy chain isotype proportions in bone marrow.

(A) Total IgE concentrations in plasma of donors.

(B) Frequency of BMPCs of different IgH isotypes among donors.

(C) Frequency of BMPCs of different IgH isotypes among clusters.

(D) Age is not a covariate that explains differences in BMPC isotype proportions among allergic and non-allergic individuals. Spearman correlation was performed.

(E) Sex is not a covariate that explains differences in BMPC isotype proportions among allergic and non-allergic individuals. Paired Friedman test was performed to identify differences.

bioRxiv preprint doi: https://doi.org/10.1101/2024.05.08.592267; this version posted May 10, 2024. The copyright holder for this preprint (which was not certified by peer review) is the author/funder, who has granted bioRxiv a license to display the preprint in perpetuity. It is made available under aCC-BY 4.0 International license.

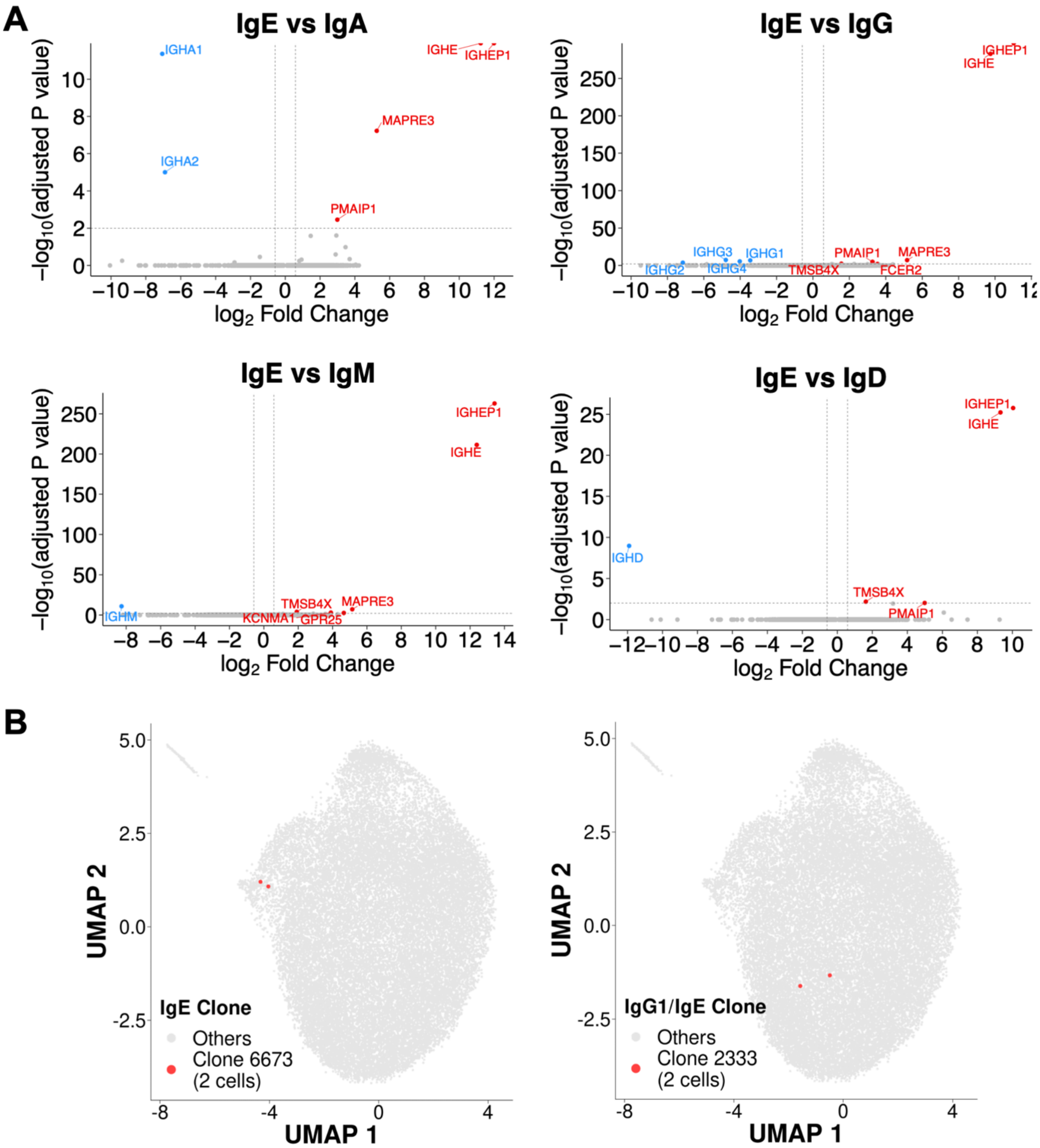

bioRxiv preprint doi: https://doi.org/10.1101/2024.05.08.592267; this version posted May 10, 2024. The copyright holder for this preprint (which was not certified by peer review) is the author/funder, who has granted bioRxiv a license to display the preprint in perpetuity. It is made available under aCC-BY 4.0 International license.

Figure S11. IgE BMPC DEGs and clonal families.

(A) Volcano plots of differentially expressed genes among IgE cells and different isotype classes.

(B) UMAP projection of clustered BMPCs, highlighting clones that include at least 1 IgE BMPC.

bioRxiv preprint doi: https://doi.org/10.1101/2024.05.08.592267; this version posted May 10, 2024. The copyright holder for this preprint (which was not certified by peer review) is the author/funder, who has granted bioRxiv a license to display the preprint in perpetuity. It is made available under aCC-BY 4.0 International license.

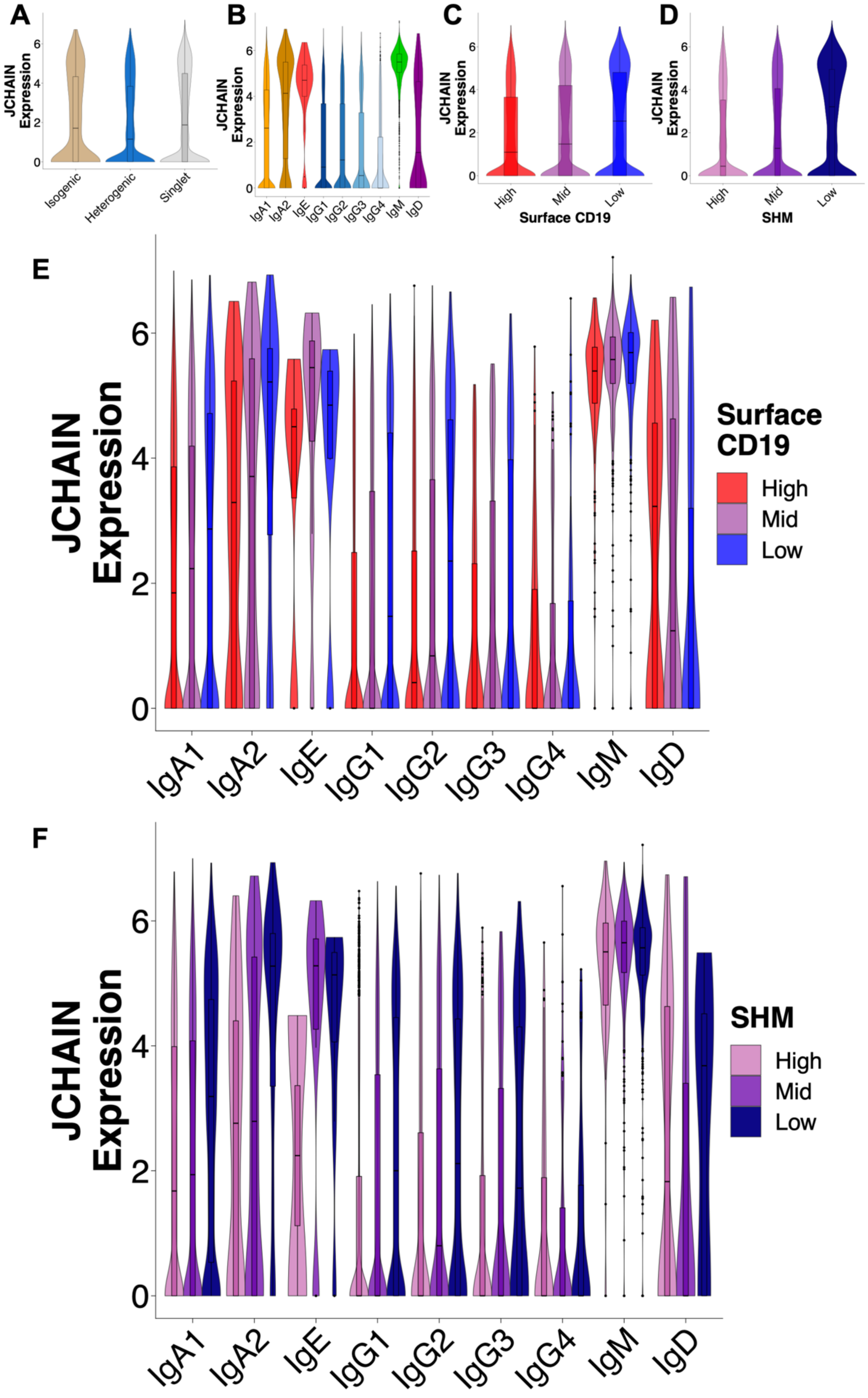

bioRxiv preprint doi: https://doi.org/10.1101/2024.05.08.592267; this version posted May 10, 2024. The copyright holder for this preprint (which was not certified by peer review) is the author/funder, who has granted bioRxiv a license to display the preprint in perpetuity. It is made available under aCC-BY 4.0 International license.

Figure S12. J chain expression among BMPCs.

(A) Expression of *JCHAIN* among clonotypes of different structures.

(B) Expression of *JCHAIN* among BMPCs of different IgH subclasses.

(C) Expression of *JCHAIN* among BMPCs belonging to different surface CD19 expression categories.

(D) Expression of *JCHAIN* among BMPCs belonging to different IgH SHM categories.

(E) *JCHAIN* expression among BMPCs of different isotypes, subdivided by surface CD19 expression categories. Sample sizes for CD19 high/mid/low are: IgA1 = 3,137 / 3,497 / 1,892; IgA2 = 165 / 218 / 141; IgE = 4 / 8 / 10; IgG1 = 2,137 / 3,411 / 3,401; IgG2 = 926 / 1,188 / 760; IgG3 = 160 / 295 / 230; IgG4 = 103 / 128 / 73; IgM = 252 / 485 / 457; IgD = 42 / 41 /30).

(F) *JCHAIN* expression among BMPCs of different isotypes, subdivided by IgH SHM categories. Sample sizes for IgH SHM high/mid/low are: nIgA1 = 3,246 / 3,341 / 1,939; nIgA2 = 107 / 204 / 213; IgE = 2 / 11 / 9; IgG1 = 2,222 / 3,725 / 3,002; IgG2 = 804 / 1,213 / 857; IgG3 = 314 / 109 / 96; IgG4 = 99 / 109 / 96; IgM = 74 / 396 / 724; IgD = 72 / 30 / 11).

bioRxiv preprint doi: https://doi.org/10.1101/2024.05.08.592267; this version posted May 10, 2024. The copyright holder for this preprint (which was not certified by peer review) is the author/funder, who has granted bioRxiv a license to display the preprint in perpetuity. It is made available under aCC-BY 4.0 International license.

Table S1. Cohort demographics.

Subject ID	Sex	Age (years)	Allergy status	Food allergies	Aeroallergy	Other allergies or intolerances	Reaction type / Time since last reaction	Total IgE (ng/mL)	Peanut-specific IgE* (kUA/L)
P0274	M	57	Non-allergic	None	No	No	n/a	258.21	n/a
P0287	M	59	Non-allergic	None	No	No	n/a	68.86	n/a
P0450†	M	27	Non-allergic	None	No	No	n/a	44.03	n/a
P0435	M	23	Allergic	Peanuts, tree nuts, sesame	Yes	No	Local, no history of anaphylaxis	705.92	n/a
P0487	F	23	Allergic	Peanuts, sesame, egg, dairy	No	Penicillins	Systemic / n.r.	505.59	69.5
P0530	F	24	Allergic	Peanuts, tree nuts, egg, dairy, mussels, oysters, clams	Yes	No	Systemic / 22 months	180.37	0.17
P0433	M	35	Allergic	Peanuts	Yes	No	Systemic / >10 years	283.29	0.37
P0520‡	F	22	Allergic	Berries, tomatoes	Yes	Latex	n.r.	82.95	0.17
P0544‡	F	22	Allergic	Peanuts	Yes	Penicillin, mosquitoes	Systemic / >2 years	601.34	>100
P0069‡§	F	34	Allergic	Peanuts, legumes, soy	No	Halothane, nickel	Systemic / 2 months	64.57	32.5
P0263‡§	F	28	Allergic	Dairy	No	No	n.r.	523.26	n/a

* Measured by ImmunoCAP.

† Populations FACS-sorted by CD19 surface expression before sequencing. Data on CD19 expression not used for analyses.

‡ VDJ rearrangements cloned into IgG1 for peanut binding testing.

§ Data not included in clustering because of strong batch effects.

n/a: Not applicable

n.r.: Not reported
